# Prevalence and impacts of self-medication in a disadvantaged setting: the importance of multi-dimensional health interventions

**DOI:** 10.3389/fpubh.2023.1176730

**Published:** 2023-07-27

**Authors:** Cuong Tat Nguyen, Hien Thu Nguyen, Laurent Boyer, Pascal Auquier, Guillaume Fond, Khanh Nam Do, Anh Kim Dang, Huyen Phuc Do, Carl A. Latkin, Roger C. M. Ho, Cyrus S. H. Ho

**Affiliations:** ^1^Institute for Global Health Innovations, Duy Tan University, Da Nang, Vietnam; ^2^Faculty of Medicine, Duy Tan University, Da Nang, Vietnam; ^3^EA 3279, CEReSS, Research Centre on Health Services and Quality of Life, Aix Marseille University, Marseille, France; ^4^Institute for Preventive Medicine and Public Health, Hanoi Medical University, Hanoi, Vietnam; ^5^Institute of Health Economics and Technology, Hanoi, Vietnam; ^6^Bloomberg School of Public Health, Johns Hopkins University, Baltimore, MD, United States; ^7^Department of Psychological Medicine, Yong Loo Lin School of Medicine, National University of Singapore, Singapore, Singapore; ^8^Institute for Health Innovation and Technology (iHealthtech), National University of Singapore, Singapore, Singapore

**Keywords:** self-medication, antibiotics, farmer, Vietnam, health interventions

## Abstract

**Background:**

Self-medication is recognized as an effective form of treatment and is increasingly encouraged to treat minor illnesses. However, misuse of self-medication leaves devastating impacts on human health and causes antimicrobial resistance. Using medication without a prescription among farmers could cause more severe effects on their health than non-farm workers since they suffer from several occupational hazards such as excessive exposure to pesticides.

**Methods:**

A cross-sectional study was conducted in 197 residents living in Moc Chau from August to September. A structured questionnaire and face-to-face were used to collecting data. The multivariate logistic model was applied to indicate associated factors with the self-medication.

**Results:**

The prevalence of self-medication among farmers was 67%. Pain relievers (66.7%) and antibiotics (32.5%) were the types of medicines that were the most commonly purchased and used without a medical prescription. Ethnics and health status also significantly affected the self-medication practice as well as the purchase and use of antibiotics. The distance to travel to a medical center and the dangerous or difficult travel, participants with arthritis or inpatient treatment had significantly associated with buying and using the medicine and antibiotics without the medical prescription of farmers.

**Conclusion:**

Our research highlights a considerably high prevalence of self-medication among farmers residing in the mountainous area of Vietnam. Individual factors such as ethnics, health status, distance to health centers, and dangerous or difficult travel were found to be related to the SM practice as well as the purchase and use of antibiotics. From that, the current study suggests interventions. For instance, official guidelines are needed to raise awareness and minimize the disadvantages of self-medication; and digital health technologies should be applied to reduce the gap in healthcare service between mountainous and other areas of Vietnam.

## 1. Introduction

Self-medication (SM), classified as a self-initiated health behavior (1), usually also refers to drug use without consulting any healthcare professionals (2, 3). Responsible SM may bring various benefits to both the individual and the healthcare system, namely the reduction in the cost of treatment, consultation time, and the workload of healthcare professionals (HCPs) (4). In recent years, SM has been recognized as an effective form of treatment and is increasingly encouraged to treat minor illnesses, most notably to prevent and manage mild symptoms of the COVID-19 or influenza seasonal (5, 6). However, abuse or misuse of SM leaves devastating impacts on the human health, particularly medication abuse some types of permitted for sale over-the-counter opioid pain medications (such as acetaminophen, acetylsalicylic acid, or caffeine), antihistamines, and laxatives (7–9), because, SM may lead to delay in diagnosis and treatment of severe medical conditions, inhibition of other diseases with similar symptoms due to the use of a non-prescription drug (10), increasing polypharmacy (11), and drug interaction (12, 13). Most importantly, unprescribed medication is listed as the most common cause of antimicrobial resistance (AMR) (14). Indeed, World Health Organization (WHO) and various researchers agree that the spread of AMR is an urgent worldwide problem requiring a global, coordinated action plan to resolve (15–19). Specifically, according to previous systematic analysis, at least 1.27 million deaths were related to AMR in 2019 and the UK Government predicted that AMR could kill 10 million people annually by 2050 without action (20, 21). In Vietnam, it was found that antibiotics can be obtained without a prescription in up to 97% of drug stores, even though the unprescribed purchase of antibiotics is illegal by the official laws (22). Several studies conducted in urban, rural, or highland areas in Vietnam found that the prevalence of SM among residents varied: around 60% in rural areas (23), 76% in urban communities (24), and 83.3% in highland areas, which was higher than that of other global regions including India (55.9–87%) (25, 26), China (25.3–31.0%) (27), or Thailand (31–35%) (28). From studies conducted in Vietnam, factors associated with SM included level of education, marital status, or income (24); mother's attitude (23); or gender, ethnic group, occupation (29).

Regarding all mentioned factors, Vietnamese farm workers were more likely to self-medicate due to low education levels, low income, traditional family dynamics, and labor-intensive job characteristics. Indeed, the rate of SM among Vietnamese farmers was higher compared to other occupations (29). Using medication without prescription among farmers could cause more severe effects on their health than non-farm workers as they are exposed to multiple occupational hazards due to excessive exposure to pesticides under a lack of government control (30), including pesticide poisoning (31), non-communicable diseases (30), infection with the soil-transmitted helminth (32), or diarrheal illness (33). Compared to other occupations, farmers also have fewer vacations and have longer working years, and more seasonal jobs such as construction workers or porters outside the harvesting season (34, 35). Uncontrollable elements such as climate change (36, 37), or animal disease outbreaks (38, 39) also contribute to the environmental pressures imposed on farmers. On top of environmental and work challenges, residents in the mountainous and remote areas face difficulties in accessing healthcare services due to poor road quality, limited transportation options, high travel costs, low budget for healthcare (40), and language barrier among ethnic minorities (41).

As an emerging public health challenge, SM among farmers has received surprisingly inadequate research attention. In Vietnam, although the general impacts of SM are investigated, very few studies have focused on the farmworker population. In this study, we chose Moc Chau, a mountainous district as our sample. It is identified with a diversity of ethnic groups and socio-demographic characteristics. This study aims to explore the prevalence of SM among farmers living in Moc Chau, Vietnam, and factors associated with SM as well as underlining potential interventions to mitigate irresponsible SM.

## 2. Materials and methods

### 2.1. Study setting and location

Data were collected from August to September 2018 in Moc Chau, a mountainous district of Son La province, Vietnam. As of 2009, the population in Moc Chau was about 152,172 (42), and the district has a total natural land area of 108,166 hectares (43). By 2014, about 33% of communes in Moc Chau have met the national standard for healthcare with 3,5 doctors/10,000 people, 13 patients/10,000 people, and one district general hospital with a total bed capacity of 150 (42).

We first prepared a list of Moc Chau districts by the number of households. After that 200 households from the list were randomly selected to participate in this study. Finally, one family member was randomly selected from each household by computer-based software to involve in a face-to-face interview and complete the questionnaire. The participants were invited to involve in the study if the following criteria were met: (1) at least 18 years old; (2) living in Moc Chau; (3) willing to take part in the study; (4) being capable of communicating with the data collectors. There were 197 respondents who agreed to join the study, and the data of this cross-sectional study was used elsewhere (44).

### 2.2. Measure and instruments

A 10-min face-to-face interview was performed. Data collectors were researchers that were well-trained to use the questionniare. Private rooms in the commune health stations were used for the interviews to maintain privacy and confidentiality. We also informed participants about the merits and drawbacks of participating in this particular study. If agreed to enroll in the study, respondents would sign written informed consent. Respondents were informed that their decision would not influence their capacity to use health services.

A structured questionnaire with four main components was designed in Vietnamese and utilized to collect data in this study, including (1) Socioeconomic characteristics; (2) Health status and psychological stress; (3) Health care utilization; and (4) Self-medication behavior characteristics. Before collecting data, this questionnaire was piloted in 20 respondents to test the language, logical order, and text. A minor change in the use of words was made to meet the participant's preferences and culture. Health staff in community health stations were not involved in the recruitment and interview process.

#### 2.2.1. Socioeconomic characteristics

The respondents provided their information about their age, gender, ethnicity, education level, marital status, average family income per month (VND), and the number of family members living in a household. After that, the average family income was exchanged to USD [1 USD ≈ 22.648 VND at August 2018 (45)].

#### 2.2.2. Health status and psychological stress

In order to assess health-related quality of life among participants within a day, we also used the EQ-VAS (visual analog scale) ranging from 0 to 100 points, labeled 0 “the worst health you can imagine” and 100 “the best health you can imagine” (46). The Kessler Psychological Distress Scale (K6) which is an abbreviated version of the K10, was used to screen for and measure the severity of mood or anxiety disorders (47). Participants were asked about their feeling of distress including nervous, hopeless, restless, depressed, effort and worthless over a period of 4 weeks prior to taking the test. These items were assessed using the 5-point Likert scale. The total score is computed by summing the points for the six experiences on the scale, severe mental illness is defined as a total score of 13 or greater. In this study, the Cronbach's alpha of this scale was good at 0.824. Furthermore, participants were asked to report their health issues that have been clinically diagnosed (Hypertension/Transient ischemic attack/Arthritis/Others).

#### 2.2.3. Health care utilization

Participants reported the number of inpatient and outpatient visits in the last 12 months. Difficulties when accessing health services, distance to travel to the medical center, and health information demands were also assessed.

#### 2.2.4. Self-medication

Participants were asked whether they had behavior purchase and use of medications without medical prescription in the last 12 months. Furthermore, participants were asked to report information related to the types of medicine that were purchased and used without medical prescription, the criteria for self-purchase and use of medications, and the main reasons to self-purchase and use medications.

### 2.3. Statistical analysis

STATA version 14.0 (Stata Corp. LP, College Station, United States of America) was used to analyze the data. A Chi-square test and the Wilcoxon Rank Sum test were used for analyzing the difference between two groups: purchasing and using medications without medical prescription and with medical prescription. To identify factors associated with SM, we applied a multivariate logistic model. A value of p < 0.05 was set for statistical significance.

## 3. Results

Table 1 shows the socio-economic characteristics of the respondents. More than half of the respondents were aged above 40-year-old (63.0%), in which 31% were from 41 to 50 years old and 32% were over 50 years old. Meanwhile, the prevalence of ethnic minorities and being married were 69.0 and 90.8%, respectively. Nearly half of farm workers in our study (42.1%) finished under secondary school and 39.5% finished secondary school. Only a small proportion (18.5%) had an education level of above secondary. Overall, most families in our sampled region had 4–5 members in their family. The average family income was 253.7 USD (SD = 211.7).

**Table 1:** Socio-economic status of participants (*n* = 197).

	**No inpatient and outpatient treatment**		**Inpatient or/and outpatient treatment**		**Total**		***p*-value**
	***n***	**%**	***n***	**%**	***n***	**%**	
**Total**	81	41.1	116	58.9	197	100.0	
**Age group**							
< 30	15	18.5	4	3.5	19	9.6	< 0.01
30–40	22	27.2	32	27.6	54	27.4	
41–50	22	27.2	39	33.6	61	31.0	
>50	22	27.2	41	35.3	63	32.0	
**Gender**							
Male	50	61.7	47	40.5	97	49.2	< 0.01
Female	31	38.3	69	59.5	100	50.8	
**Ethnic**							
Kinh	24	29.6	37	31.9	61	31.0	0.74
Others	57	70.4	79	68.1	136	69.0	
**Education**							
Under secondary	34	42.5	48	41.7	82	42.1	0.55
Secondary	34	42.5	43	37.4	77	39.5	
Above secondary	12	15.0	24	20.9	36	18.5	
**Marital status**							
Married	75	92.6	103	89.6	178	90.8	0.50
Others	6	7.4	12	10.4	18	9.2	
	**Mean**	**SD**	**Mean**	**SD**	**Mean**	**SD**	***p*** **-value**
**Age (years)**	42.4	12.0	46.6	11.4	44.9	11.8	0.01
**Number of family members**	4.5	1.8	4.6	1.9	4.5	1.8	0.68
**Family income (USD)**	244.0	206.8	259.9	215.6	253.7	211.7	0.52

Table 2 indicates the health status and health care utilization of farmers living in Moc Chau. Overall, only a small proportion of participants suffered from health issues, in which the most common issue was arthritis (20.3%). The majority (90.9%) of participants had normal psychological state, but a considerable proportion (9.1%) had severe mental illnesses. The most prevalent difficulties in accessing health services among our participants were far distance (15.2%) and dangerous and difficult travel (11.2%). The mean EQ-VAS score was 67.8 (SD = 15.5), while the average distance from home to the nearest medical facility was 4.1 km (SD = 5.9).

**Table 2:** Health status and health care utilization of farmers living in Moc Chau.

	**No inpatient and outpatient treatment**		**Inpatient or/and outpatient treatment**		**Total**		***p*-value**
	***n***	**%**	***n***	**%**	***n***	**%**	
**Health issues**							
Hypertension	5	6.2	21	18.1	26	13.2	0.02
Transient ischemic attack	7	8.6	26	22.4	33	16.8	0.01
Arthritis	11	13.6	29	25.0	40	20.3	0.05
Others	4	4.9	18	15.5	22	11.2	0.02
**Number of morbidities**							
Do not have any disease	59	72.8	59	50.9	118	59.9	< 0.01
Have one disease	19	23.5	31	26.7	50	25.4	
Have more than one disease	3	3.7	26	22.4	29	14.7	
**Psychological distress**							
Normal	75	92.6	104	89.7	179	90.9	0.62
Severe mental illness	6	7.4	12	10.3	18	9.1	
**Difficulties in accessing health services**							
Far distance	16	19.8	14	12.1	30	15.2	0.14
Not have vehicles	5	6.2	2	1.7	7	3.6	0.10
Dangerous and difficult travel	12	14.8	10	8.6	22	11.2	0.16
Others	1	1.2	5	4.3	6	3.1	0.23
**Availabilityy of village health workers**	74	91.4	109	94.0	183	92.9	0.48
	**Mean**	**SD**	**Mean**	**SD**	**Mean**	**SD**	***p*** **-value**
**EQ-VAS (score range: 0–100)**	70.7	15.1	65.9	15.5	67.8	15.5	0.04
**Distance to travel to a medical center (km)**	4.7	6.4	3.7	5.5	4.1	5.9	0.22

Table 3 presents the SM of farmers in the mountainous area of Vietnam. More than two-thirds of the respondent reported that they purchased medications without a medical prescription (67%). About one-third of participants (32.5%) purchased and used antibiotics without a medical prescription. Most common factors in criteria for self-purchase and use of medication without medical presciption included: drugs given by unofficial medicine sellers (86.4%) and reused a previous prescription (9.9%). As for the reasons for self-purchase, 59.9% of the participants self-purchased medications as they believe that their condition was mild, compared and about a quarter (22%) supposed that they do not have enough time to go to the hospital. Other common reasons included far distance from the nearest medical center (18.2%) and personal experience (17.4%). The most common types of medicines for personal use and self-purchase were pain relievers (66.7%) and antibiotics (32.5%).

**Table 3:** Self-medication of farmers in a mountainous area in Vietnam.

**Characteristics**	**No inpatient and outpatient treatment**		**Inpatient or/and outpatient treatment**		**Total**		***p*-value**
	***n***	**%**	***n***	**%**	***N***	**%**	
**Behavior purchase and use of medications without medical prescription**	56	69.1	76	65.5	132	67.0	0.60
**Types of medicine were purchased and used without medical prescription**							
Pain relievers	41	73.2	47	61.8	88	66.7	0.17
Antibiotics	24	29.6	40	34.5	64	32.5	0.47
Sustenance	9	11.1	19	16.4	28	14.2	0.30
Functional food	2	2.5	4	3.5	6	3.1	0.69
Others	7	8.6	12	10.3	19	9.6	0.69
**Criteria for self-purchase and use of medications**							
Remembered the name of the medication	5	8.9	8	10.5	13	9.9	0.76
Reused a previous prescription	1	1.8	12	15.8	13	9.9	0.01
Drugs given my unofficial medicine sellers	54	96.4	60	79.0	114	86.4	< 0.01
Available medicine at home	1	1.8	8	10.5	9	6.9	0.05
**Reason to self-purchase and use medications**							
Experienced	3	5.4	20	26.3	23	17.4	< 0.01
It was an emergency	3	5.4	5	6.6	8	6.1	0.77
Supposed that disease is mild	41	73.2	38	50.0	79	59.9	0.01
Did not have time to go to the hospital	8	14.3	21	27.6	29	22.0	0.07
Did not have money to affort healthcare services	2	3.6	10	13.2	12	9.1	0.06
Distance is too far to the nearest medical center	11	19.6	13	17.1	24	18.2	0.71

Table 4 describes the associated factors with the self-treatment of Vietnamese farmers in the mountainous area. Respondents who came from ethnics other than Kinh had 2.16 times higher tendency or purchasing and using medication without medical prescription and 3.12 times higher tendency of purchasing and using antibiotics without medical prescription. Respondents who suffered from arthritis also had 2.63 times higher likelihood of self-purchasing and using antibiotics. The respondents who had longer distance to travel to a medical center further away were more likely to buy and use the medicine without a prescription, specifically 1.15 times higher in comparison with nearer distances (95% CI = 1.03; 1.28). The purchase and use of antibiotics without medical prescription increased 3.60 times among farmers with dangerous and difficult travel and 4.46 times among those who had inpatient treatment, or no outpatient treatment in the past 12 months.

**Table 4:** Association factors with self-medication of Vietnamese farmers in Moc Chau.

**Factors**	**Purchase and use medications without medical prescription**		**Purchase and use antibiotics without medical prescription**	
	**OR**	**95% CI**	**OR**	**95% CI**
**Gender (vs. male)**				
Female	1.35	0.66; 2.77	1.28	0.61; 2.67
**Age**	0.98	0.95; 1.02	0.98	0.94; 1.01
**Education (vs. under secondary)**				
Secondary	1.45	0.67; 3.14	1.44	0.65; 3.16
Above secondary	0.78	0.28; 2.13	0.6	0.20; 1.78
**Ethnic (vs. Kinh)**				
Others	2.16^*^	0.91; 5.09	3.12^***^	1.34; 7.28
**Health issues**				
Transient ischemic attack (yes vs. no)	0.76	0.28; 2.05	0.5	0.18; 1.39
Arthritis (yes vs. no)	1.68	0.68; 4.15	2.63^**^	1.09; 6.34
Hypertension (yes vs. no)	0.95	0.33; 2.77	1.05	0.34; 3.19
**Distance to travel to a medical center (km)**	1.15^**^	1.03; 1.28	0.96	0.88; 1.04
**Difficulties in accessing health services**				
Far distance (yes vs no)	0.26^**^	0.08; 0.89	0.73	0.21; 2.50
Not have vehicles (yes vs no)	1.42	0.19; 10.68	1.93	0.26; 14.03
Dangerous and difficult travel (yes vs no)	1.66	0.50; 5.49	3.60^**^	1.15; 11.28
**Times to visit of village health workers**	0.94	0.82; 1.08	1.06	0.92; 1.21
**Using medical services (vs. no inpatient and outpatient treatment)**				
Inpatient treatment	1.64	0.43; 6.18	4.46^**^	1.27; 15.70
Outpatient treatment	0.87	0.39; 1.94	0.99	0.42; 2.33
Inpatient and outpatient treatment	0.8	0.32; 1.98	1.73	0.68; 4.41

^***^p < 0.01.

^**^p < 0.05.

^*^p < 0.1.

## 4. Discussion

The results indicated that SM was a common practice among Moc Chau residents, in which antibiotics were one the most common means of SM. The prevalence of SM among farmers (67%) from our study was higher than that of other Asian countries such as China (45.4%, 2015) (48), India (7.7%, 2014) (49), Nepal (59%, 2002) (50), and Pakistan (51%, 2008) (51). Notably, this figure was extremely higher in European countries, namely Germany (27.7%, 2013) (52) and Greece (23.4%, 2002) (53). Overall, ~½ of the participants suffered from health issues, in which the most common issue being arthritis (20.3%) and transient ischemic attack (16.8%). The most prevalent difficulties in accessing health services among our participants were far distance and dangerous and difficult travel. The most common reasons for self-purchase were misbelief about the severity of their disease and inadequate time to seek healthcare. Factors associated with higher tendency of self-purchase were ethnic group, arthritis condition, distance from home to health facility and traveling conditions.

Our results showed the insignificant difference between the rate of people who used health services utilization and who did not use (58.9 and 41.1%, respectively). This difference, though not substantial, was recorded through self-report and contributed to health system performance assessment considering the existing gap in data globally and especially in developing countries (54, 55). Moreover, the utilization of health services was not only associated with service availability but also with the preference, trust, and socioeconomic characteristics of the community (56, 57). Health care-seeking behavior also varied between demographics and income levels. For example, countries with higher income average tend to have higher rates of outpatient service utilization such as 84% in Singapore (58) or 64.1% in Taiwan (59), while developing countries often record high SM rates, as suggested by evidence from Vietnam (55, 57). Several socio-economic (such as financial condition) and health status factors, barriers to accessing health services, as well as ease of unregulated antibiotic access, have been shown to be significantly correlated with SM and AMR (60). In this study, perceiving the disease as mild (59.9%), not having time to go to the hospital (22%), and choosing medicine based on personal experience (17.4%) were three primary reasons of SM among Moc Chau farmers and were also found in previous studies (48, 61). Other associated factors found were history of the disease, access to the pharmacist (62), cost-efficiency, or unavailability of health services (63, 64). About 32.5% of the participants in our study purchased antibiotics without a prescription. Indeed, 91% of antibiotics sold in Vietnam were without a prescription (65), which was even higher than that of countries in Asia such as Thailand (37.4%) (66), Indonesia (45%) (67), India (54.2%) (68), Korea (46.9%) (69), and Nepal 50.7% (70). The high prevalence of purchasing antibiotics in Vietnam might be attributed to (1) lax enforcement of selling and using antibiotics (71) and (2) lack of awareness about the side effects of antibiotic resistance (72). Therefore, stricter regulations and constant information should be provided to the general population.

Our results suggested a correlation between distance to health facilities and unprescribed medication. Understandably, in disadvantaged regions, health facilities tend to be inaccessible due to difficult transportation and low-quality roads, unlike in contrary to urban and city settings, where transportation is generally more developed and convenient (41). On the other hand, among respondents who have received inpatient services in health facilities, the rate of antibiotic SM was higher than those who never received onsite healthcare. This pattern can be explained by the exposure to antibiotics of previous patients. The common mentality shared by those who were once hospitalized is to reuse previous prescriptions for similar illnesses, and thus they tended to re-purchase the antibiotics once prescribed. Moreover, in rural and disadvantaged areas in Vietnam, residents relied greatly on mouth-to-mouth and unverified advice on medication without consulting a qualified health official. Similarly, we found that ethnic minorities tend to use antibiotics without prescription more than Kinh people did. Vietnamese ethnics have limited availability and access to healthcare services and health information, and thus tend to buy medicine at the most convenient drug stores (40). This behavior pattern can be attributed to a lack of trust in the national healthcare system and a lack of awareness about the risks involved in irresponsible SM.

Notably, farmers having arthritis tended to buy antibiotics without prescription nearly three times higher than those without that disease (OR = 2.63, 95% CI = 1.09–6.34), which suggested a common practice in Vietnam where antibiotics were applied for most diseases regardless of their necessity (73). A previous study found that patients with arthritis were more likely to develop and used SM as an immediate response instead of undergoing a long process of hospitalization (74). In addition, arthritis shared many similarities and was often confused with inflammation (75) and infection. As a result, farmers who had low education levels and lacked antibiotics awareness might misperceive antibiotics as a treatment for inflammation. Moreover, our study revealed that medicine sellers consulted and recommended the medicine to 86.4% of participants, which was higher than the findings of Selvaraj et al. conducting in India (38%) (61) and Jember et al. conducting in Northwest Ethiopia (46%) (76). Indeed, in Vietnam, the regulations for the distribution of antibiotics and other medications in general among personal medicine facilities were rather inconsistent and lenient. As a result, many private facilities, even unqualified pharmacies, have been able to import and sell medicines without following official guidances. More interestingly, these facilities tended to locate in rural areas as the legal scene in these communities are substantially less strict than in crowded or center regions. Understandably, as medications were easily purchased without medical prescription, residents in rural areas, which were farm workers in our study, tended to consult pharmacists and thus increased the rate of self-medicaiton in Moc Chau. This result showed the role of unqualified pharmacists in increasing the misuse of antibiotics and pointed to an urgent need for stricter regulations in the distribution of antibiotics to local pharmacists (77).

According to the guidelines of WHO (78), despite several advantages of SM, an array of potential risks of SM has also been indicated. Specifically, the main potential risks need to worth noting that (1) lead to delay/incorrect diagnosis of medical conditions; (2) existed the risk of double medication (if using the same active substance is under another name) or harmful drug interactions with concomitantly taken other drugs; (3) wastage of resources, increased resistance of pathogens and causes serious health hazards such as the risk of drugs dependency/abuse as well as increased drug resistance (78). From our study, several implications were drawn. First, authorities and healthcare managers should develop and publish an official pharmacy guidance, where distribution of certain medication is stated clearly and implemented strictly accordingly. At the same time, the government should adjust policies on unprescribed medication purchases to implement heavier financial fines and severe punishments, such as degree suspension. In addition, guidelines should be launched to encourage responsible SM and minimize unnecessary side-effects due to lack of information, not only to the general public but also to local pharmacists. Second, the government should consider technology applications in diagnosis and drug prescription such as telemedicine, mHealth, digital health, and eHealth services to adapt to the increasing demand for healthcare services and narrow the gap in diagnosis and treatment among areas in Vietnam. Given the high levels of adaptability to online interventions among citizens, medication purchase and pharmacy consults should also be conducted online. This approach can resolve at least two reasons for SM, namely far distance to nearest health facility and not having time to go to the hospital. Lastly, awareness-raising campaigns should be implemented more intensively in rural areas and especially for ethnic minorities. These groups are the most vulnerable to self-purchase without medical prescription due to their lack of knowledge about medicine and negative effects of mis-purchasing. Raising awareness of minority groups should be prioritized and delivered through user-friendly measures considering the low level of adaptability among this group. For instance, the global campaign “World Antibiotic Awareness Week (WAAW)” is celebrated each November and achieved significant efficiency in improving awareness and understanding of AMR as well as encouraging best practices among the general population, health workers, and policymakers to limit the further emergence and spread of AMR globally (79). To sum up, regardless of implementation means, these activities need to be conducted regularly and over a long period of time to maintain and amplify their impacts.

This study has some limitations that should be considered. First, the self-reported questionnaire might cause recall bias. Secondly, because this study employed a sample of cross-sectional observations, it is not able to produce the findings for the long-term trends of SM among farmers in the mountainous area of Vietnam. Secondly, despite our study was excluded all health staff in the recruitment and interview process, however, there was a chance that someone in the farmers' families is a healthcare professional, which may lead to bias in the findings of this study. Finally, we recruited participants in Moc Chau districts only, which may limit generalization to other populations in mountainous areas of Vietnam. However, the results of this study still can be used as evidence for identifying risk factors in SM as well as providing insights into SM practices and then creating the base for a larger study.

## 5. Conclusions

Our study contributes to the current understanding of the prevalence of SM among farmers in remote areas in Vietnam. Our research highlights a considerably high prevalence of SM among farmers residing in the mountainous area of Vietnam. Ethnics, health status, distance to health centers, and dangerous or difficult travel were risk factors that affected the SM practice as well as the purchase and use of antibiotics. The current study suggests interventions for addressing the SM situation. Official guidelines are needed to raise awareness and minimize the disadvantages of SM. Besides, digital health technologies should be applied to reduce the gap in healthcare service between mountainous and other areas of Vietnam.

## Data availability statement

The raw data supporting the conclusions of this article will be made available by the authors, without undue reservation.

## Ethics statement

The study was conducted in accordance with the Declaration of Helsinki and approved by the Institutional Review Board of Hanoi Medical University (code: 20/HMUIRB). Informed consent was obtained from all subjects involved in the study.

## Author contributions

CN, LB, PA, and GF: conceptualization. HN: data curation and formal analysis. KD and AD: investigation. CN, HN, and HD: methodology. KD: project administration. KD and AD: resources. CN: software and visualization. CN, RH, and CH: supervision. HN and KD: validation. CN and HN: writing—original draft. CN, HD, LB, PA, GF, CL, RH, and CH: writing—review and editing. All authors contributed to the article and approved the submitted version.
